# Effect of Acute Exposure to Moderate Altitude on Muscle Power: Hypobaric Hypoxia vs. Normobaric Hypoxia

**DOI:** 10.1371/journal.pone.0114072

**Published:** 2014-12-04

**Authors:** Belén Feriche, Amador García-Ramos, Carmen Calderón-Soto, Franchek Drobnic, Juan G. Bonitch- Góngora, Pedro A. Galilea, Joan Riera, Paulino Padial

**Affiliations:** 1 Department of Physical Education and Sport, University of Granada, Granada, Spain; 2 High Performance Centre of Sierra Nevada, High Sport Council, Granada, Spain; 3 Department of Sport Physiology, Grup d’Investigació en el Rendiment i la Salut de l’Esportista d’Alt Nivell Esportiu del Centre D'Alt Rendiment, High Sport Council, Barcelona, Spain; Emory University, United States of America

## Abstract

When ascending to a higher altitude, changes in air density and oxygen levels affect the way in which explosive actions are executed. This study was designed to compare the effects of acute exposure to real or simulated moderate hypoxia on the dynamics of the force-velocity relationship observed in bench press exercise. Twenty-eight combat sports athletes were assigned to two groups and assessed on two separate occasions: G1 (n = 17) in conditions of normoxia (N1) and hypobaric hypoxia (HH) and G2 (n = 11) in conditions of normoxia (N2) and normobaric hypoxia (NH). Individual and complete force-velocity relationships in bench press were determined on each assessment day. For each exercise repetition, we obtained the mean and peak velocity and power shown by the athletes. Maximum power (P_max_) was recorded as the highest P_mean_ obtained across the complete force-velocity curve. Our findings indicate a significantly higher absolute load linked to P_max_ (∼3%) and maximal strength (1RM) (∼6%) in G1 attributable to the climb to altitude (*P*<0.05). We also observed a stimulating effect of natural hypoxia on P_mean_ and P_peak_ in the middle-high part of the curve (≥60 kg; *P*<0.01) and a 7.8% mean increase in barbell displacement velocity (*P*<0.001). No changes in any of the variables examined were observed in G2. According to these data, we can state that acute exposure to natural moderate altitude as opposed to simulated normobaric hypoxia leads to gains in 1RM, movement velocity and power during the execution of a force-velocity curve in bench press.

## Introduction

During ascent to higher altitude, the partial pressure of oxygen (O_2_) in the air gradually diminishes and this reduces the arterial partial pressure of O_2_ leading to tissue hypoxia [1]. It is known that endurance performance is compromised at hypoxic enviroments and mean reductions in VO_2max_ of 6% per 1000 m of ascent have been described [2]. For short-duration high-intensity activities lasting less than 1 min, the predominant energy source is phosphorylation and non-oxidative production of ATP [3]. Given that explosive performance is not aerobic dependent, short-explosive actions should not be impaired by altitude. In fact, it was observed during the Mexico City Olympics Games in 1968 (at 2240 m) in sprint events [4].

Peronnet et al. [4] proposed that air density decrement at altitude (∼3% reduction for each 305 m rise [5]) diminish the energy cost of running at high velocities without impairing energy availability. The reduction in external resistance to movement [6] and/or the modified muscle recruitment pattern due to increased anaerobic metabolism [7], [8], could be related to this reduced energy cost and thus improve performance in rapid actions such as throws, jumps or blows [5], [6]. However, strength and resistance training at altitude have been scarcely addressed in the scientific literature. Some studies have related the severe hypoxia of high altitude (>5500 m) to muscle deterioration and reduced muscular function [9], [10] and power [11], including a loss of up to 15% lean mass [12], along with a reduced strength gain (−6.4%) compared to that produced in conditions of normoxia for the same training exercise [13].

In contrast, the effect of exposure to a real moderate altitude (2000–3000 m asl) on muscle power has not yet been adequately addressed, despite this being the altitude most athletes select for training. Recently, Scott et al. did not find an effect of moderate and high acute simulated hypoxic stimulus (fraction of inspired oxygen [F_i_O_2_] of 0.16 and 0.13) during a resistance high intensity in back squat and dead lift exercises on force and power measurements [14]. Conversely, Chirosa et al. [15] reported an improvement in the force-velocity curve for half back squat in 5 recreational athletes after rapidly ascending to an altitude of 2320 m. Using the load at which maximum power was achieved in normoxia, in acute moderate hypoxia, 4% gains in velocity and 7% gains in power were produced (*P*<0.05). Likewise, performance of 27 elite swimmers was assessed in normoxia and at acute moderate altitude, and a small yet non-significant improvement was recorded in 50 m front crawl time, attributed to an increase (+3.2%; *P*<0.05) in velocity during the first 15 m [16]. Also in swimming, for a given velocity in 400 m (freestyle), Mercadé et al. [17] noted changes in swimming technique (ie, a 2.4% increase in cycle frequency) induced by acute exposure to real moderate altitude, although these changes could not be correlated with the physiological alterations that accompanied the ascent.

In general, this effect of altitude on the mechanical components of an athlete’s movement has not been dealt with in detail. The available literature has mainly addressed the hypothesis of the reduced energy cost improving isolated high speed running times [4] through a reduction in aerodynamic resistance in direct proportion to rapid displacement velocities in individuals (eg, running) or objects (eg, hurling) [5], [6]. In contrast, we focused our study on the relationship hypoxia-movement (eg, leg displacement) rather than on its consequence (eg, the starting velocity of a kicked ball), opening up a new line of investigation that considers the effects of both air composition and its resistance. Given that athletes attend training camps at real altitude, this study aims to compare the effect of a reduction in barometric pressure and/or changes in air composition on the capacity to develop explosive efforts in bench press following real versus simulated acute exposure to moderate hypoxia.

## Materials and Methods

### Ethics statement

Written informed consent to participate in this study was obtained from each subject. For individuals younger than 18 years, authorization was obtained from their parents or legal guardian. The study protocol was consistent with the principles outlined in the Declaration of Helsinki and was approved by the Ethics Committee of the University of Granada.

### Experimental design

A repeated measures design was employed with two independent groups (G1 and G2). Subjects in both groups were tested on two occasions separated by a rest period of 48 h. Subjects in G1 were tested first in conditions of normoxia (N1) and then following their ascent to the High Performance Centre of Sierra Nevada (Spain) at 2320 m asl to determine the effect of conditions of hypobaric hypoxia (HH). Subjects in G2 were first tested in conditions of normoxia (N2) and then after exposure to simulated normobaric hypoxia (NH) at the High Performance Center of Sant Cugat (NE Spain). Simulated NH was achieved by breathing a mixture of air impoverished in oxygen (15.7% FiO_2_) corresponding to an altitude of 2300 m. Complete force–velocity relationships for only-concentric bench press (BP) were obtained on each assessment day in each subject. For each test, the variables power and velocity using each load, maximal strength (1RM) and the load linked to maximum power were calculated and subjected to intra- and intergroup comparisons.

### Subjects

Twenty-eight male Olympic combat sports athletes (wrestling n = 16, judo n = 7 and taekwondo n = 5) voluntarily participated in the study. All subjects were in their competition period and had participated in national and international competitions at least since the year prior to the study outset. Sports experience was >8 years and the athletes trained for a mean of 10–18 h per week. None of the subjects were taking drugs, medication, or dietary supplements known to influence physical performance. Descriptive characteristics of the subjects are provided in Table 1. A test for unpaired data detected no significant differences between the two groups.

**Table 1:** Descriptive characteristics of the subjects (mean ± SD).

	n	Age (years)	Height (cm)	Body mass (kg)	1RM (kg·kg^−1^)
G1	17	22.82±3.83	175.56±8.35	79.53±13.59	1.04±0.16
G2	11	22.45±5.03	177.45±10.00	72.73±13.03	1.05±0.19
P value			*P*>0.05		

### Methodology

Subjects visited the laboratory after refraining from intense physical activity for at least 48 h. Before the tests they undertook a standard warm up protocol consisting of 15 min of activation, joint mobility and stretching exercises and a further warm up in which they performed two sets of 5 repetitions at maximum velocity in BP using a 20 kg weight (∼20–30% 1RM). The rest interval between sets was 3 min.

Individual force–velocity relationships were determined via a progressive load test in only-concentric phase BP. The starting load was 20 kg and this was increased by 10 kg per set until the individual’s 1RM. One set of 2 to 4 repetitions was performed per load. The recovery period between sets was 3 min for velocities ≥1 m•s^−1^ or 5 min for velocities <1 m•s^−1^. All the tests were performed in a Smith’s machine in which the barbell was attached to both ends, with linear bearings on two vertical bars allowing only vertical movements.

Subjects commenced the test by supporting the barbell with arms extended above the chest. From this position, the barbell was lowered in a continuous movement until it was around 5 cm from the chest and this position maintained for 3 s. Next, the subjects were instructed to perform a purely concentric action as fast as possible to return to the starting position. No bouncing, arching of the back or launching of the barbell was allowed. Trained spotters were present when high loads were lifted to ensure safety. Subjects were verbally encouraged to successfully complete each exercise.

Mechanical variables were recorded using a linear position transducer (Real Power Pro Globus, Codgne, Italy linked to a Tesys 400) and Ergo System 8.5 software. The system was fixed to the barbell such that the cable was vertically displaced and informed of the barbell trajectory at a frequency of 1000 Hz. For each repetition, we obtained a mean and maximum value of the velocity (V) and power (P). Only the best repetition for each load in terms of the greatest mean power generated (P_mean_) was entered in the subsequent analysis. We established as maximum power (P_max_), the highest P_mean_ recorded across the full curve. The load corresponding to P_max_ for each subject was obtained from the load-P_mean_ polynomic equation constructed using data for the exercise sets comprising the whole test.

Subjects assigned to the NH test wore a silicon mask connected to an oxygen-depleting respiratory system (HYP100, Hypoxic Inc System, Shekou Shenzhen, China) from 5 min before warm up to test completion.

### Statistical analysis

Data are presented as mean ± standard deviation (SD). The normality of data distribution was checked using the Shapiro-Wilk test. The influence of hypoxia exposure for each group (pre vs. post) on each dependent variable was assessed with paired t-tests. Performance absolute differences on each group (HH-N1 vs. NH-N2) were used to compare hypobaric vs. normobaric hypoxia effects. Wilcoxon and Mann-Whitney U tests were used when data was not normally distributed. In that case, confidence intervals were estimated following Hodges-Lehman’s procedure. Furthermore, mean power values were extrapolated from the force-velocity curve at fixed loads (20, 40, 60, 80 and 100% 1RM) relative to the corresponding 1RM in each condition (N1, N2, HH and NH). The magnitude of the main differences between comparisons was also expressed as standardized mean difference (Coheńs d effect size; ES). The criteria to interpret the magnitude of the ES were as follows: <0.2 = trivial, 0.2–0.6 = small, 0.6–1.2 = moderate, 1.2–2.0 = large, 2–4.0) very large and >4 = extra large [18]. Significance was set at *P*≤0.05. All statistical tests were performed using SPSS version 20.0 (SPSS, Chicago, IL).

## Results

Intragroup comparisons revealed a moderate increment in 1RM (+5.73%; ES = 0.3) and a small increase in the overall load corresponding to P_max_ (+3.29%; ES = 0.2) compared to normoxia values in G1 attributable to the subjects’ ascent to a moderate altitude, whereas no differences were detected in G2 (Table 2). When the effect of two hypoxia conditions were compared (effect of G1 vs effect of G2), natural hypoxia only was linked to a higher RM (*P* = 0.01; ES = 1.1) together a moderate increase to the load corresponding to P_max_ near to signification (*P* = 0.09, ES = 0.69).

**Table 2:** Intra group comparisons of results linked to maximum power and maximum dynamic force.

	1RM (kg)		P_max_ (W·kg^−1^)		LoadP_max_ (kg)		%RMP_max_	
	G1	G2	G1	G2	G1	G2	G1	G2
**N**	83.33	76.36	5.29	4.87	48.27	43.09	58.25	56.80
	±15.43	±16.29	±0.60	±0.51	±8.03	±8.61	±4.16	±5.34
**H**	88.00	75.45	5.36	4.82	49.73	42.64	56.94	57.44
	±16.56	±18.64	±0.64	±0.47	±8.16	±8.72	±4.35	±5.87
***P***	0.004	0.588	0.244	0.402	0.040	0.631	0.355	0.765

*1RM = 1 repetition maximum; P_max_ = maximum power; LoadP_max_ = absolute load linked to maximum power; %RMP_max_ = percentage of 1RM linked to maximum power; N = conditions of normoxia; H = conditions of hypoxia; G1 = group 1; G2 = group 2; P = p-value.*

Tables 3 and 4 provide the values of P_mean_, P_peak_ and V_mean_ for the different loads in G1 and G2 respectively. Comparisons of HH vs. N1 revealed significant increases at HH in P_mean_, P_peak_ and V_mean_ from 60 kg, except in the P_peak_ at 80 kg in which the significance was border liner (*P*<0.08). In contrast, the same comparison in G2 indicated no significant differences for any of the loads examined.

**Table 3:** Mean power, peak power and mean velocity recorded in conditions of normoxia versus hypobaric hypoxia.

			P_mean_ (W·kg^−1^)		P_peak_ (W·kg^−1^)		V_mean_ (m·s^−1^)	
Load	N	Cond	Mean ± SD	*P*	Mean ± SD	*P*	Mean ± SD	*P*
20	17	N	3.82±0.51	0.882	10.10±1.26	0.289	1.39±0.11	0.810
		HH	3.83±0.46		10.27±1.09		1.39±0.14	
30	17	N	4.63±0.56	0.895	10.25±1.21	*0.003*	1.16±0.13	0.776
		HH	4.64±0.50		10.88±1.15		1.16±0.12	
40	17	N	5.15±0.52	0.082	10.86±1.37	0.984	0.99±0.13	0.089
		HH	5.03±0.51		10.86±1.36		0.97±0.13	
50	17	N	5.01±0.68	0.263	10.38±1.56	0.894	0.79±0.17	0.357
		HH	5.14±0.69		10.34±1.25		0.81±0.15	
60	17	N	4.57±1.08	0.004	9.04±1.72	0.016	0.61±0.19	0.010
		HH	4.82±1.02		9.55±1.26		0.64±0.18	
70	13	N	4.54±1.16	0.041	8.73±1.42	0.051	0.52±0.17	0.048
		HH	4.77±1.16		9.31±1.74		0.55±0.17	
80	11	N	3.82±1.29	0.001	7.64±1.72	0.076	0.41±0.16	0.001
		HH	4.20±1.25		8.30±1.46		0.45±0.16	
90	7	N	3.65±0.71	0.025	6.91±0.82	0.049	0.36±0.09	0.024
		HH	3.93±0.82		7.62±0.62		0.38±0.09	

*(Data display truncated with n<5). Load (kg); P_mean_ = mean power; P_peak_ = peak power; V_mean_ = mean velocity; n = number of values included in the analysis; Cond = test conditions. N = normoxia; HH = hypobaric hypoxia. P = p-value.*

**Table 4:** Mean power, peak power and mean velocity recorded in conditions of normoxia versus normobaric hypoxia.

			P_mean_ (W·kg^−1^)		P_peak_ (W·kg^−1^)		V_mean_ (m·s^−1^)	
Load	n	Cond	Mean ± SD	*P*	Mean ± SD	*P*	Mean ± SD	*P*
20	11	N	3.69±0.27	0.595	8.81±0.93	0.959	1.249±0.14	0.669
		NH	3.75±0.47		8.79±1.00		1.263±0.13	
30	11	N	4.47±0.27	0.807	8.96±0.90	0.414	1.045±0.14	0.673
		NH	4.45±0.44		9.16±0.94		1.038±0.13	
40	11	N	4.67±0.48	0.768	8.58±1.41	0.498	0.840±0.17	0.487
		NH	4.63±0.47		9.01±1.99		0.829±0.16	
50	11	N	4.46±0.77	0.989	7.91±1.48	0.627	0.654±0.17	0.986
		NH	4.46±0.85		7.79±1.74		0.655±0.18	
60	9	N	4.32±1.28	0.889	7.67±1.96	0.643	0.553±0.19	0.804
		NH	4.29±0.76		7.49±1.71		0.546±0.15	
70	7	N	4.04±1.10	0.605	7.41±1.11	0.658	0.456±0.13	0.704
		NH	3.97±1.08		7.33±1.02		0.451±0.13	
80	6	N	3.08±1.37	0.152	6.20±1.69	0.839	0.312±0.15	0.153
		NH	3.30±1.28		6.22±1.52		0.336±0.15	

*(Data display truncated with n≤5). Load (kg); P_mean_ = mean power; P_peak_ = peak power; V_mean_ = mean velocity; n = number of values included in the analysis; Cond = test conditions. N = normoxia; NH = normobaric hypoxia. P = p-value.*

Table 5 display a second analysis in which means for P_mean_, P_peak_, V_mean_ and V_peak_ recorded for light loads (20 to 50 kg) and heavy loads (60 to 100 kg) were compared within groups. This comparison revealed a small effect (ES = 0.20) from of natural hypoxia (G1) on all the variables recorded using heavy weights (*P*<0.01) and only a trivial (ES<0.20) but significant on the V_peak_ generated using light weights. In contrast, under conditions of simulated hypoxia (G2) no gains were produced in any of the variables examined.

**Table 5:** Power and velocity recorded for different loads in conditions of normoxia versus hypoxia.

				P_mean_ (W·kg^−1^)		P_peak_ (W·kg^−1^)		V_mean_ (m·s^−1^)		V_peak_ (m·s^−1^)	
Group	Load (kg)	n	Cond	Mean ± SD	*P*	Mean ± SD	*P*	Mean ± SD	*P*	Mean ± SD	*P*
**G1**	20–50	68	N	4.65±0.77	0.847	10.39±1.35	0.062	1.08±0.26	0.895	1.86±0.47	0.004
			HH	4.66±0.75		10.59±1.22		1.08±0.26		1.89±0.47	
**G1**	60–100	55	N	4.04±1.25	<0.001	8.10±1.73	0.002	0.48±0.20	<0.001	0.83±0.27	<0.001
			HH	4.32±1.22		8.58±1.73		0.51±0.19		0.88±0.28	
**G2**	20–50	44	N	4.32±0.61	0.977	8.56±1.23	0.513	0.95±0.27	0.906	1.56±0.51	0.644
			NH	4.33±0.66		8.69±1.53		0.95±0.27		1.56±0.52	
**G2**	60–100	27	N	3.71±1.28	0.865	6.92±1.72	0.659	0.42±0.19	0.783	0.70±0.27	0.633
			NH	3.73±1.13		6.85±1.56		0.42±0.18		0.70±0.27	

*P_mean_ = mean power; P_peak_ = peak power; V_mean_ = mean velocity; V_peak_ = peak velocity; n = number of values included in the test; Cond = test conditions. N = normoxia; NH = normobaric hypoxia. P = p-value.*

**Figure 1:**
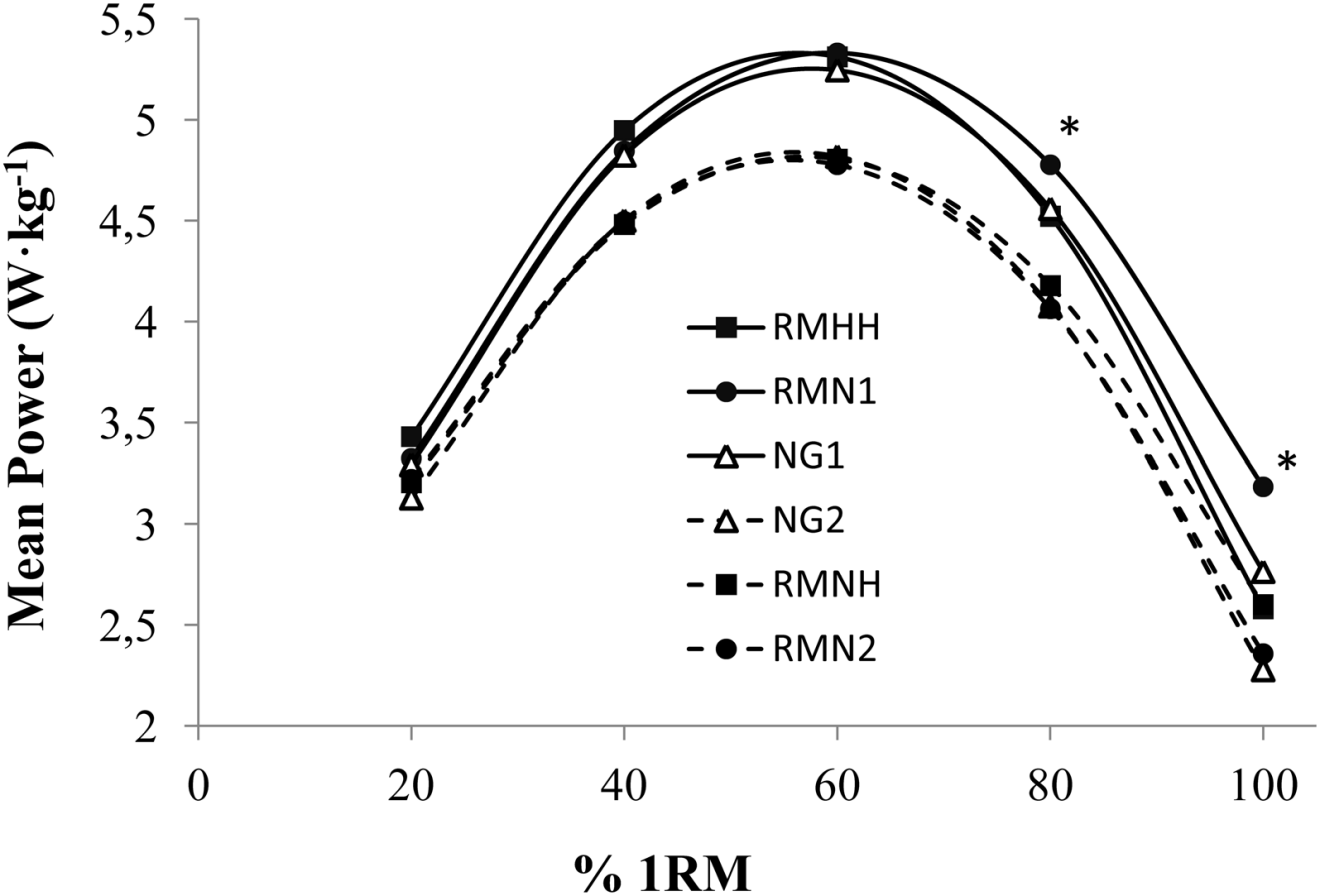
Mean power output for different % 1RM. RMHH = HH curve constructed using the 1RM for hypobaric hypoxia; RMN1 = HH curve constructed using the 1RM for normoxia; NG1 = normoxia curve for G1; NG2 = normoxia curve for G2; RMNH = NH curve constructed using the 1RM for normobaric hypoxia; RMN2 = NH curve constructed using the 1RM for normoxia; * = Significant differences between NG1 and RMN1 (*P*<0.01).

Finally, Figure 1 shows the P_mean_ values recorded in the two groups for different 1RM percentages (20, 40, 60, 80 and 100% 1RM), adjusted in each case to the “normoxia” 1RM or “hypoxia” 1RM. We observed no differences in the P_mean_ values recorded in G1 (N1 vs. HH) or G2 (N2 vs. NH) at any % 1RM (i.e. hypoxia power curve for 1RM of hypoxia vs normoxia power curve for 1RM of normoxia). However, when the two curves were expressed relative to the normoxia 1RM, powers in G1 were overestimated for HH vs. N1 at 80 to 100% 1RM (*P<*0.01). No appreciable changes in lower % 1RM values in G1 were detected nor were any differences detected for any of the loads examined in G2. Accordingly, the % 1RM corresponding to the P_max_ recorded in conditions of HH was higher (*P* = 0.005; ES = 0.8) when the load was adjusted to the 1RM recorded in conditions of normoxia or N1 (60.05±3.88% 1RM), and differed from the value obtained when the load was adjusted to the 1RM observed in conditions of hypobaric hypoxia HH (56.94±4.35% 1RM). In contrast no such shift was produced in G2 (*P* = 0.395).

## Discussion

As the main finding of our study, we observed an effect on the behavior of the force-velocity curve of acute exposure to a moderate altitude compared to negligible effects of simulated conditions of hypoxia. Contrary to conditions of normoxia or simulated hypoxia, real hypoxia resulted in a faster displacement velocity of the barbell and a higher P_mean_ for a given workload in BP, which led to a higher load corresponding to P_max_ (+3.29%) and a gain in 1RM (+5.73%) (*P*<0.05). Thus, real altitude effect improves the velocity of a loaded movement and it seems that this effect is more linked to the reduced density of air than to diminished availability of O_2_. However, the interaction effect between air pressure decrements and O_2_ availability was not studied and could potentially be responsible for the results. One of the novelties of this study was that these changes were detected in a basic training exercise rather than in complete competition activities or through the analysis of the trajectory of a thrown or hit object. According to the results of our study, the loads used for training at normal altitude cannot be translated to training programs performed at higher altitude. This is especially true, given the relevance of locating and assessing the behavior of maximum power for rapid force training prescription.

The aim of this study was to discriminate the influence of oxygen availability and/or barometric pressure reduction on power and velocity performance during a whole force-velocity curve. Although previous research has investigated whether a period of resistance training performed while breathing normobaric hypoxic air can induce muscle hypertrophy [19], [20], power and velocity of the movement have not been frequently examined or controlled. Only Scott et al. [14], monitored power and force trends over 5 sets of 5 repetitions at 80% of 1RM under acute moderate and high normobaric hypoxia, showing no differences from normoxic conditions. However, the exposition to a real altitude improves performance in short-duration actions such as throws, jumps, or launching objects [5], [6]. A recent study performed with 18 young male swimmers, from the Junior Spanish National team, revealed an average improvement of muscular P_peak_ and V_peak_ of 12.07±1.81% and 6.56±1.22% respectively in overloaded squat jump after ascent to a moderate altitude [21]. In agreement with this finding, the data observed for BP exercise in conditions of HH (G1) indicate a higher V_peak_ than that produced in normoxia lifting both light (20–50 kg; *P*<0.01) and heavy weights (60–100 kg; *P*<0.001) (see Table 5). However, according to mean power and velocity values attained with light weights, the magnitude of the effect reached <60 kg in V_peak_ was trivial, which compromises the practical value of this result. We consider that lack of changes in power and velocity with low weights may be inherent to the nature of the exercise used in the study. A traditional BP executed at maximum velocity comprises a breaking phase at the end of the movement, likely due to the increased activity of the antagonist musculature and reduced actions of agonist muscles, which avoids losing the grip on the barbell and the stopping of movement [22]. This deceleration diminishes as the load lifted increases [23], [24], such that when deceleration constitutes a high % in the repetition (∼52% for light loads), large changes in V_peak_ and V_mean_ are needed for differences to be detected in the repetition. In contrast, the lower % deceleration produced with heavy loads (∼23%) means that small changes in these velocities can lead to significant changes in the whole repetition [23]. This could justify the P_mean_ and V_mean_ increments only at loads ≥60 kg in real altitude and the lack of changes when reporting mean velocity and power values attained with light loads (Tables 3–5; Fig. 1). In agreement with the results of G2, Scott et al. did not find a moderate simulated hypoxia effect (F_i_O_2_ = 16%) on mean and peak force and power variables during a period of high intensity resistance training [14].

Despite of limited evidence, there is increasing research examining the physiological effects of hypoxia on resistance training. A recent review of resistance training adaptation mechanisms described a relationship between metabolic stress induced by the build-up of H^+^ or by low O_2_ saturation (SaO_2_) and the recruitment of additional fast twitch muscle fibers [8]. Then, one possibility is that ascent in altitude induce an anaerobic morpho-functional profile that improves the recruitment of high threshold motor units leading to perform the movement faster. But in an opposite way, the lack of changes in peak and mean power in G2 breathing air impoverished in O_2_ (FiO_2_ 15.7%) questions this idea. It is known that hypoxia and insufficient brain oxygenation reduce the electrical activity of neurons [25], [26]. In this sense, some studies have linked diminished oxygenation of the prefrontal cortex in conditions of acute hypoxia (FiO_2_ 13%, ∼3500 m) to electromyographic abnormalities in the muscles involved in 10-s sprints [27]. However, for isolated short-burst brief actions such as those assessed in this study (<5 s plus 3–5 min rest), this effect is not observed in moderate or high normobaric hypoxia (16–13% FiO_2_) [14], been needed SaO_2_ levels <82% (which happened above altitudes of 3500 m asl) [26]. In contrast, real altitude, combining hypoxia and air density reductions, seems to improve the force-velocity relationship in G1. An additional benefit of the natural vs. simulated hypoxia has been also concluded in other studies after chronic exposure. Millet et al, showed mean improvement in power output of 4.1% at real altitude vs. 1% with artificial hypoxia. However, the main physiological mechanisms remain unclear [28], [29], and in any case, they were not related with the changes of the motor skill that could be associated with altitude.

No differences in P_max_ under normoxic conditions were obtained between the experimental groups. However, we observed a moderate increase in the effect of real altitude respect the simulated in the overall load linked to P_max_ (effect of G1 vs. effect of G2; ES = 0.7). Thus, the P_max_ in normoxia and/or simulated hypoxia was recorded for around 3% less load than when the exercise was executed under real hypoxia. Other authors have also described that acute natural hypoxia increases the load corresponding to P_max_ by 5.6% in half squat [15]. However, the increased 1RM at real altitude in comparison to the simulated hypoxic conditions (5.58%; ES = 1.1), determines that this effect disappears when the P_max_ load is expressed as a % of the corresponding 1RM. Figure 1 in the results shows plots of P_mean_ vs. % 1RM in the different study conditions. The P_mean_ values obtained differ according to the 1RM considered in each case when comparing N, HH or NH. Under real hypoxia, 1RM underwent an increase close to 6% with respect to the value recorded in conditions of N (*P*<0.01), which was not observed for NH. By adjusting the P_mean_ curve using as reference the 1RM recorded for N, the power curve is shifted upwards and to the right such that P_mean_ is overestimated for loads >60% 1RM compared to the curve obtained using the 1RM observed for HH. This finding has several practical applications that coaches need to consider when prescribing power training at altitude. For example, if we use the same loads for training under conditions of normoxia as we use for HH, we will be developing “more” power at loads above the load linked to P_max_, and “less” power using loads below that linked to P_max_, assuming the exercise is always executed at maximum velocity.

One of the limitations of this study is that the design does not allow us to determine whether or not there are interaction effects between the change in air density and the low O_2_ pressure of the air breathed by the subject on the power recorded. On that purpose, a third experimental condition at real altitude breathing a 21% FiO_2_ should have been included. Also, this study has been conducted after an acute exposure to hypoxia and the differences between real and normobaric simulated hypoxic conditions might be greater after a period of muscular power training (altitude camps normally are three weeks long). From our results, we can conclude that breathing air with reduced O_2_ pressure (by 15.7%) or being naturally exposed to moderate altitude had different effects on the variables examined. It would seem that as for conditions of normoxia, movement velocity and load adjustment should both be considered when planning an altitude strength training protocol since these are the real indicators of the true load of the work undertaken. Since improved power has been linked to better neuromuscular characteristics [30], [31], our results indicate the change in air resistance produced when moving to a higher altitude could promote these adaptations, allowing for the development of greater power, unlike the situation for NH. The increasing tendency of change shown by 1RM and the load linked to P_max_ in the G1 group suggests a need to adjust the training load during weight training sessions at altitude to avoid reducing the muscle stimulus. Thus, by adjusting strength training loads we could help avoid the loss of muscle mass and/or inter- and intra-muscle coordination that are common after periods of altitude training [6]. There is a need for longitudinal studies that will provide information on muscle behavior following training in real conditions of hypoxia. This will enable athletes to add to the benefits of altitude those arising from training focused on the exercise technique and its rapid execution in sports modalities other than endurance.

## Conclusions

Our findings indicate that acute exposure to moderate hypoxia leads to gains in velocity and power when executing a force-velocity curve in BP. Unlike the case for simulated conditions of altitude, at real altitude, the load at which P_max_ was recorded was 3.3% higher, and means power values above those of normoxia for the rest of the middle-high zone of the curve were observed for a similar absolute load. We therefore recommend not transferring loads calculated for strength training in conditions of normoxia to real altitude. A displacement of some 6% of 1RM in conditions of HH will reduce the training stimulus in greater proportion as loads approach that corresponding to the maximum (1RM), movement velocity being the best indicator of workload. The stimulating effect of hypoxia on the speed of loaded movement in this study happened at real altitude and seems more related to reduced air density than to reduced inspired O_2_, although the main mechanism remain unclear.
